# Feasibility of a Self-Managed, Video-Guided Exercise Program for Community-Dwelling People with Stroke

**DOI:** 10.1155/2021/5598100

**Published:** 2021-04-27

**Authors:** Katharine Scrivener, Jessica Sewastenko, Alexandra Bouvier-Farrell, Katherine MacDonald, Tegan Van Rijn, Joshua Tezak, Nicholas Mandis, Sherrie Love

**Affiliations:** Department of Health Professions, Macquarie University, Ground Floor 75 Talavera Rd, Australia

## Abstract

**Background:**

Ongoing rehabilitation after stroke is limited. Using video-guided exercises, which are implemented with a self-management approach, may be a way to facilitate ongoing exercise in the home environment.

**Objectives:**

To investigate the feasibility of a video-guided exercise program, implemented with a self-management approach for people with stroke.

**Methods:**

A phase I, single-group, clinical trial. The study comprised two phases: in phase one, four weeks of the program was supported by weekly supervised sessions and in phase two, four weeks of the program was completed without direct supervision. Demographic information was recorded at baseline. Adherence and adverse events were self-reported via a logbook. Acceptability was measured through a purpose-built scale. Physical performance, physical activity, and exercise self-efficacy were measured at baseline and 4 and 8 weeks.

**Results:**

Sixteen people with stroke were recruited; however, 14 commenced and completed the study. Adherence during the supervised phase was 3.3 hours per week and 2.3 hours per week during the self-directed phase. There were no adverse events. Most participants indicated that the program was easy to use (92%) and would recommend the program to others (86%). Walking speed improved over the duration of the program (mean difference -0.12 m/s, 95% CI -0.22 to -0.02, *p* = 0.02). Self-efficacy and physical activity did not change over the duration of the program.

**Conclusion:**

The findings support the feasibility of a video-guided exercise program for people with stroke. Further research to confirm the effectiveness of this intervention to improve physical function is warranted.

## 1. Introduction

In Australia, people with stroke do not commonly have the opportunity to undertake long-term rehabilitation. After discharge from hospital, they attend an average of 11 sessions of physiotherapy and occupational therapy [1]. Furthermore, their overall physical activity profile is low, with 63-90% of waking hours spent in sedentary activities [2]. People with stroke want to access goal-directed and meaningful physiotherapy [3]. They want to practice regularly and complete everyday tasks [4], and they have shown interest in accessing programs delivered remotely [5]. A major clinical challenge is determining how to deliver self-management effectively in people with stroke in order for them to carry out ongoing physical activity.

Self-management is a process in which an individual is supported in managing their own health condition. Self-management has been validated as an effective tool in many chronic health conditions [6], particularly when it is focused on supporting behaviour change and increasing self-efficacy [7]. There has been little investigation of self-management to increase physical activity for people with stroke [8]. This is likely due to the difficulties in working with this population, due to the nonmotor impairments commonly present with stroke. Despite these limitations, clinical practice guidelines recommend that people with stroke should have opportunities for self-directed practice, and it is proposed that self-management should be included within stroke care [9, 10].

A limited number of self-management programs for people with stroke living in the community have been developed. These include remotely delivered advice, education about physical activity, and activities to enhance the skills required for self-management of physical activity [11]. The investigations show this type of program to be feasible and acceptable to individuals with stroke. This study extends this concept by offering a structured, video-guided exercise program, TASK, to support people with stroke in self-managing their exercise.

It is possible that, with complex conditions such as stroke, a more structured approach is required to maximise the chances of self-management being effective. This structured approach includes resources (TASK), training, ongoing support, and enhancing self-efficacy for sustained self-management of exercise.

The specific research aims were

1. to determine if it is feasible to deliver TASK embedded in a self-management approach in terms of safety, adherence, acceptability, and cost
2. to determine if TASK, delivered in two stages—TASK supervised practice and TASK self-directed practice—improves outcomes (physical activity, walking, and self-efficacy)

Our hypothesis was that it would be feasible to deliver TASK with a high adherence rate and acceptability. The intervention would provide a low-cost solution when compared to ongoing institution-based sessions, with no significant safety issues or adverse events.

## 2. Materials and Methods

### 2.1. Study Design

A phase I, single-group, clinical trial was conducted. People with stroke were recruited from a private community-based rehabilitation clinic in Sydney, Australia. Participants undertook the TASK program. First, they undertook 4 weeks of physiotherapist-supervised practice at the rehabilitation clinic. Second, participants undertook 4 weeks of self-directed practice at home. Outcomes were feasibility and clinical outcomes (physical activity, walking, and self-efficacy), collected at baseline, week 4, and week 8, by a researcher blinded to the research questions. The study was approved by the Macquarie University Human Research Ethics Committee (approval number 520183224478). This study was prospectively registered on 6/8/2018 (registration number ACTRN12618001316291p).

### 2.2. Participants

People with stroke were included if they were an adult, have been discharged from rehabilitation, and could walk at >0.4 m/s with/without an aid. They were excluded if they could not understand verbal/written English or did not have Internet access at home. Information such as age, gender, side of hemiparesis, cognition (measured via the MMSE), and time poststroke was collected to describe the sample.

### 2.3. Intervention

From week 1 to 4, participants undertook TASK physiotherapist-supervised practice, for 30 minutes once a week at the rehabilitation centre. The TASK program involves the practice of five everyday tasks: sitting, standing up, standing, stepping, and stepping up. Each task is practised for approximately 5 minutes. The program is set to music. Each task has a video that demonstrates the exercise, highlights the correct technique, and notes common errors. Participants received a resource package including an electronic copy of the program (via an app or website), exercise mat (for the floor and table), and exercise step. The exercise floor mat is designed to be a visual cue to remind people to exercise within the home and to guide equipment placement. It also provides stepping targets. During the four weeks of supervised training, the physiotherapist supervising the program implemented a self-management approach, in which they taught the participant how to monitor their own technique, how to progress or regress each exercise, and how to alter the dose. The main aim of these sessions was to equip the participant with the skills needed to continue the program safely with increasing independence in the home. As soon as the participant was familiar with the program and safe to complete aspects, they were asked to commence self-directed practice of TASK at home.

From week 5 to 8, participants undertook TASK self-directed practice, for 30 minutes three times a week at home. The physiotherapist contacted each participant weekly via the participant's preferred contact method (video call, phone, or email). This contact was designed to provide coaching, motivation, troubleshoot issues, etc.

### 2.4. Outcome Measures

Feasibility of delivering the intervention was measured in terms of safety, adherence, acceptability, and cost. For safety, data on near misses and adverse events was collected. Adverse events consisted of minor events including muscle soreness, and major events will include falls and injuries requiring medical care. Participants completed a logbook documenting their adherence to the program and minor or major adverse events. For acceptability, participants were asked four questions: (i) Were you satisfied with the TASK program? (ii) Was TASK program easy to use? (iii) Did you enjoy using the TASK program? (iv) Would you recommend this program to others?

Clinical outcomes were physical activity, walking, and self-efficacy. Physical activity was measured using the Incidental and Planned Exercise Questionnaire (IPEQ), a self-reported measure of physical activity [12]. Walking was measured using the 5-metre Walk Test (5mWT). Self-efficacy was measured using the Exercise Self-Efficacy Scale, a 10-item scale that rates confidence in exercise [13]. The maximum score which can be achieved on this scale is 40. Cost for the program was calculated based on the physiotherapist's usual fees and the cost of the equipment.

### 2.5. Data Analysis

Data was analysed using SPSS version 25. Demographic data were reported via descriptive statistics. Adherence data was calculated from week 0 to week 4 and then week 4 to week 8 and reported as mean values for the supervised and self-directed phases. Results from the four acceptability questions were reported as frequencies comparing time points week 4 and week 8. Paired *t*-tests were used to analyse within-group change over time for the clinical outcome measures at time points week 0 to week 4, week 4 to week 8, and week 0 to week 8. Differences are reported as a mean and 95% confidence interval.

## 3. Results

### 3.1. Flow of Participants during the Study

Sixteen participants were recruited to the study; however, two individuals withdrew before baseline data was collected with one individual reporting underlying health concerns and one lacking time to complete the intervention as the reasons for their withdrawal. Therefore, 14 participants completed the intervention and were assessed at baseline and week four, respectively. At 8 weeks, 13 of the participants completed all the reassessments.

### 3.2. Demographic Data

The mean age of the participants was 57 years (SD 14.2). There were large variations in timeframes poststroke (mean = 35.3 months, SD = 30.9). Participants' mean cognition score on the MMSE was 28/30 (SD 1.5, range 26-30, *n* = 9). The majority of the participants were male (64%). Mean initial walking speed was 0.87 m/s (SD 0.5), and 71% of participants walked without a walking aid. There were 9 individuals with right-sided hemiparesis (64%), 4 with left (29%), and one participant with no hemiparesis reported (7%).

### 3.3. Adherence

Adherence of participants to the TASK program between week 0 and week 4 (supervised phase) averaged 3.3 hours per week (SD 2.1, *n* = 13) and between week 4 and week 8 (self-directed phase) averaged 2.3 hours per week (SD 1.5, *n* = 7). This indicates that adherence was higher during the phase that was supervised in comparison to the self-directed phase.

### 3.4. Acceptability

The majority of participants were either satisfied or very satisfied (77%) by the TASK program at the conclusion of the study. Almost all the participants reported the program to be easy or very easy to use (92%); however, one participant did report that it was difficult to use. All but one participant indicated that the TASK program was enjoyable. The majority of the participants indicated that they would either recommend or strongly recommend (86%) the TASK program to others.  Table 1 includes the detailed results of the four acceptability questions.

### 3.5. Cost

The 8-week TASK program as conducted in this study cost a total of $737 Australian Dollars per participant. This cost includes provision of equipment and therapist costs for the supervised and remote sessions.

### 3.6. Safety

No adverse events, minor or major, were reported by the participants in this study.

#### 3.6.1. Outcomes

*(1) Walking Speed*. The average walking speed of participants at baseline was 0.87 m/s (SD 0.5, *n* = 14). At 4 weeks, the average walking speed was 0.92 m/s (SD 0.4, *n* = 14) and at 8 weeks 1.02 m/s (SD 0.6, *n* = 13). There was a significant difference between initial and final walking speed (mean difference -0.12 m/s, 95% CI -0.22 to -0.02, *p* = 0.02). See Table 2 for further details.

*(2) Self-Efficacy*. The mean scores at week 0, week 4, and week 8 were 28.6 (SD 4.5, *n* = 14), 30.6 (SD 5.2, *n* = 14), and 30.8 (SD 6.8, *n* = 13), respectively, out of a total score of 40. See Table 2 for further details.

*(1) Physical Activity*. At baseline, the mean physical activity score was 23.7 (SD 16.3, *n* = 14). This only varied slightly at 4 weeks (mean 25.0, SD 17.0, *n* = 14) and 8 weeks (23.5, SD 14.8, *n* = 13). See Table 2 for further details.

## 4. Discussion

This study investigated the feasibility of a video-guided exercise program, delivered with a self-management approach for community-dwelling people with stroke. Participants found the program acceptable, reporting that it was enjoyable and they would recommend it to others. Adherence to the program was good; however, it did decrease in the self-directed phase (3.3 hours compared to 2.3 hours per week). This was anticipated as during the supervised phase, participants could be reminded in person in comparison to the remote support. There were no adverse events reported in this study, suggesting that a self-directed video-based program can be implemented safely.

Offering exercise programs facilitated by technology and videos has been previously investigated for people with stroke [14, 15] and in wider populations [16–18]. Video-guided exercise has been demonstrated to impact adherence but not outcomes over more traditional paper or booklet programs [18]. Furthermore, it has been found feasible for people with health conditions to access videos on an iPad, though not all liked using them [17]. It seems that personal preference is important with some people wanting to use technology whilst others prefer more traditional approaches. There may however be significant advantages of using technology to guide exercise programs. These include an increased ability to communicate a correct technique (both verbally and visually) and provide ongoing motivation and tips. Based on these benefits, it is warranted to pursue video-guided exercises in rehabilitation.

In previous studies that have implemented technology for people with stroke, many did not include a supervised phase consisting of familiarisation sessions. This perhaps explains why reported adherence levels have been low [14]. It could be hypothesised that people with stroke may need more initial support to access technology than people with other health conditions. A positive initial experience with technology has been shown to be highly influential to the ongoing success of a technology-based intervention [19]. This study implemented initial face-to-face sessions with a self-management approach aimed at upskilling participants and transitioning them into a self-directed phase. Similar to this study, White and colleagues employed familiarisation in their study of tablet-facilitated rehabilitation after stroke [15]. Participants in this study reported finding this beneficial and overall that the technology kept their rehabilitation interesting [15].

The current study did have limitations which need to be considered when interpreting the findings. Firstly, as a phase 1, feasibility study, the sample size for this study was small and diverse in characteristics. Secondly, the study utilised a survey which was purpose-built and allowed for only answers to be selected with no further input of the participant. This restricted the ability to collect in-depth information regarding each participant's experience and perspective of the TASK program. Having more information about the participants' experience with the program could have been valuable in informing future research and clinical practice.

## 5. Conclusion

In conclusion, the findings of this study suggest that a video-guided exercise program, implemented with a self-management approach, is a feasible and accepted intervention for people with stroke. Future research should be conducted in a larger trial with a control group to determine if this type of exercise program can impact physical outcomes and self-efficacy.

## Figures and Tables

**Table 1:** Detailed results of the 4 acceptability questions at 4- and 8-week time points (*n* = 13).

Question	Time point	Frequency of each response (*n*)				
		Very unsatisfied	Unsatisfied	Neutral	Satisfied	Very satisfied
Were you satisfied with the TASK program?	Week 4	0	0	2	6	5
	Week 8	0	0	3	6	4

		Very difficult	Difficult	Neutral	Easy	Very easy
Was TASK program easy to use?	Week 4	0	0	1	9	3
	Week 8	0	1	5	6	1

		Very unenjoyable	Unenjoyable	Neutral	Enjoyable	Very enjoyable
Did you enjoy using the TASK program?	Week 4	0	0	3	8	2
	Week 8	0	0	1	12	0

		Strongly not recommend	Not recommend	Neutral	Recommend	Strongly recommend
Would you recommend this program to others?	Week 4	0	0	0	8	5
	Week 8	0	1	0	7	5

**Table 2:** Change in outcome measures comparing baseline to week 4 and week 8.

Time point	Participants (*n*)	Measure	Mean difference	95% CI	*p* value
Weeks 0-4	14	Walking speed	-0.04 m/s	-0.13 to 0.05	0.31
	14	Self-efficacy	-1.93	-4.74 to 0.89	0.16
	14	Physical activity	-1.27	-9.32 to 6.78	0.74

Weeks 4-8	14	Walking speed	-0.91 m/s	-0.24 to 0.06	0.20
	13	Self-efficacy	-0.08	-2.98 to 2.83	0.96
	14	Physical activity	2.42	-3.47 to 8.31	0.39

Weeks 0-8	13	Walking speed	-0.12 m/s	-0.22 to -0.02	0.02
	13	Self-efficacy	-2.77	-6.44 to 0.90	0.13
	13	Physical activity	1.93	-5.74 to 9.60	0.59

## Data Availability

Data is available from the authors on request.
